# Impact of caller’s degree-of-worry on triage response in out-of-hours telephone consultations: a randomized controlled trial

**DOI:** 10.1186/s13049-019-0618-2

**Published:** 2019-04-11

**Authors:** Hejdi Gamst-Jensen, Erika Frishknecht Christensen, Freddy Lippert, Fredrik Folke, Ingrid Egerod, Mikkel Brabrand, Janne Schurmann Tolstrup, Lau Caspar Thygesen, Linda Huibers

**Affiliations:** 1 Emergency Medical Services Copenhagen, Copenhagen University, Copenhagen, Denmark; 2 Center for Prehospital and Emergency Research, Department of Clinical Medicine, Aalborg University, Aalborg, Denmark; 3 Clinic of Emergency Medicine and Department of Anaesthesiology and Intensive Care, Aalborg University Hospital South, Aalborg, Denmark; 4 Department of Cardiology, Copenhagen University Hospital, Gentofte, Denmark; 5 Department of Intensive Care, Copenhagen University Hospital, Rigshospitalet, Copenhagen, Denmark; 6 Department of Emergency Medicine, Odense University Hospital, Odense, Denmark; 7 Department of Emergency Medicine, Hospital of South West Jutland, Esbjerg, Denmark; 8 National Institute of Public Health, University of Southern Denmark, Copenhagen, Denmark; 9 Research Unit for General Practice, Aarhus, Denmark

**Keywords:** Out of hours medical care, Randomized controlled trial, Triage

## Abstract

**Background:**

Telephone triage entails assessment of urgency and direction of flow in out-of-hours (OOH) services, while visual cues are inherently lacking. Triage tools are recommended but current tools fail to provide systematic assessment of the caller’s perspective. Research demonstrated that callers can scale their degree-of-worry (DOW) in a telephone contact with OOH services, but its impact on triage response is undetermined. The aim of this study was to investigate the association between call-handlers’ awareness of the caller’s DOW and the telephone triage response.

**Methods:**

A randomized controlled trial at a Danish OOH service using telephone triage with quantitative analyses and qualitative process evaluation. Prior to contact with a call-handler, callers were asked to rate their DOW on a five-point scale. Calls were randomized to show or not show DOW on the call-handlers’ screens. Triage response (telephone consultation or face-to-face consultation) was analysed using Chi-square tests. Process evaluation incorporated a quantitative and qualitative assessment of intervention implementation and fidelity.

**Results:**

Of 11,413 calls, 5705 were allocated to the intervention and 5708 to the control group. No difference in number of face-to-face consultations was detected between the two groups (OR 1.05, 95% CI 0.98 to 1.14, *p* = 0.17). The process evaluation showed that call-handlers did not use the DOW systematically and were reluctant to use DOW.

**Conclusion:**

Awareness of DOW did not affect the triage response, but this finding could reflect a weak implementation strategy. Future studies should emphasise the implementation strategy to determine the effect of DOW on triage response.

**Trial registration:**

Registration number, Clinicaltrials.gov NCT02979457.

**Electronic supplementary material:**

The online version of this article (10.1186/s13049-019-0618-2) contains supplementary material, which is available to authorized users.

## Background

Telephone triage is a widely used gatekeeping function of assessing the level of urgency of the patient’s health problem and directing the flow of out-of-hours (OOH) services [1]. In telephone triage, a call-handler makes clinical decisions based on the dialogue with the caller. Clinical decision-making in telephone triage is particularly challenging due to the lack of visual cues, and might be complicated by individual human factors related to the caller or the call-handler, such as vague symptom description, non-professional communication or fixation error [2–6]. Triage tools are recommended in telephone triage, but these are mainly based on criteria and symptom intensity [7], and are criticized for failing to incorporate the patient perspective and context [8, 9].

Patients’ motives for contacting OOH services range from information seeking to a cry for help in true urgency [10–12]. Nonetheless, patients’ perceptions of their illness or injury are rarely assessed and documented during OOH telephone triage contacts [13, 14]. Research shows that call-handlers favour biomedical problems [14, 15] and that open-ended questions are not commonly used during telephone triage contacts [5, 14]. When restricting the communication to closed-ended biomedical questions, exploration of the patient perspective may become superficial with potential loss of valuable information. A feasibility study on self-rated degree-of-worry (DOW) demonstrated that patients volunteered more medically relevant information when they were asked about their DOW [16], and studies on DOW show an association between DOW, illness perception and health outcome [16, 17].

Call-handlers have expressed fear of making the wrong decision by underestimating the illness severity and facing public scrutiny [9, 18], thereby constraining themselves to a more conservative triage pattern and referring more patients to the hospital [19, 20]. DOW could be a valuable addition to decision tools in telephone triage, supporting clinical decision-making. However, knowledge of the effect of call-handlers’ awareness of DOW on the triage response is yet to be explored. Thus, the aim of this study was to investigate the association between call-handlers’ awareness of the caller’s DOW and the telephone triage response. The predefined hypothesis was that awareness of DOW would lead to fewer face-to-face consultations.

## Methods

### Design

We performed a pragmatic randomized controlled study providing the call-handlers with information of the caller’s DOW in a computer assisted randomization and following for triage response. The study is reported in accordance with the CONSORT statement [21]. The intervention was assessed with both a quantitative and qualitative process evaluation.

### Setting

The acute care system within the Capital Region of Denmark can be reached by calling the Emergency Medical Service Copenhagen, where two different telephone numbers act as access points to pre-assessment and triage. The telephone number 1–1-2 receives emergency calls for assumed life-threatening injury/illness (130,000 calls per year) and the medical helpline 1813 (MH1813) with the phone number 1–8–1-3 receives calls for any other medical request including referral to an emergency department (ED) for acute non-life-threatening illness/injury. At MH1813 registered nurses or physicians triage the caller to either self-care, own general practitioner (GP) during office hours, face-to-face consultation at the ED, home visit, direct hospitalisation, or ambulance dispatch. The MH1813 handles approximately one million calls per year of which roughly 40% are triaged to either self-care or contact with their own GP. Triage and determination of urgency is guided by a criterion-based triage tool, which consists of three main categories: somatic illness, somatic injury, and psychiatric illness. Each category is sub-divided into a number of options depending on symptom localisation and severity. The triage tool was developed locally without validation [22].

### Data collection and variables

Data was collected from January 24 to February 9, 2017. The telephone answering machine included a message, with the option to decline participation by pressing ‘2’ (Additional file 1). Eligible callers were Danish speaking patients or their close relatives/friends who agreed to participate. Participants received an automated telephone questionnaire to elicit the caller’s DOW before the call-handler responded to the call. They were asked to describe their relation to the patient and rate their DOW on a 5-point scale by responding to the following question: “How worried are you about your problem on a scale from one to five, where one is minimum worry and five is maximum worry?” Staff at the MH1813 were informed of the study at staff meetings, in newsletters and on the regional website.

Data on the triage response were collected from the computerized patient records at the Emergency Medical Service Copenhagen along with age, gender and reason for encounter (RFE) based on the main categories of the triage tool (i.e. somatic illness, somatic injury, psychiatric illness, or other). Telephone triage response was categorized as: 1) telephone consultation (on-the-spot self-care advice by the call-handler or advice to contact the own GP), 2) face-to-face consultation (referral to an ED, physician home-visit, hospitalisation, or ambulance dispatch), or 3) other (other guidance, answer on blood test, case summary after home visit).

### Intervention

The intervention consisted of informing the call-handlers of the caller’s DOW. According to protocol, call-handlers in the intervention group were visually informed of the caller’s DOW prior to taking the call. The call-handlers were instructed to use the DOW at their discretion. To gain the most possible visibility, DOW was planned to show below the personal identification number on the screen displaying the computer aided dispatch system template (Additional file 2).

### Randomization

Each call was given a unique session identification number with a time stamp and was randomly assigned to the control or intervention group as per computer generated randomization schedule (1:1). After randomization the call-handler in the intervention group was visually informed of the DOW, while the call-handler in the control group was blinded.

### Sample size

Sample size was calculated based on an effect size of 2.5% change in triage response, power of 80% and significance level of 5%. Approximately 40% of calls ended with a telephone consultation [22]. Thus, a study population of 4232 in both intervention and control group would be needed. To allow incomplete variables, we aimed to include 5000 participants per group.

### Intervention process evaluation

A process evaluation is used to identify components of an intervention that are effective and under which conditions [23]. The process evaluation was carried out by HGJ, performing spot-checks of call-handlers’ awareness of the ongoing data collection and the location of DOW on their screens twice daily for five days. This awareness was calculated by the number of informed call-handlers as a percentage of all active call-handler stations. The awareness was deemed satisfactory if the call-handlers were aware of the data collection taking place and also knew where to locate DOW on their screens. The intervention quality-and-integrity [23] was assessed by HGJ through semi-structured interviews with a convenience sample of 13 call-handlers three weeks after termination of the data collection. The interview guide (Additional file 3) focused on the awareness of DOW and perception of the usefulness of DOW in telephone triage. The audio-recorded interviews were listened through three times by HGJ while taking notes and a thematic analysis was used to analyse the interviews. This method entails familiarisation with the data, coding, searching for themes, reviewing themes and defining and naming the themes [24].

### Statistical methods

We applied intention-to-treat analysis, blinded to group allocation. The primary outcome was telephone triage response (telephone consultation vs. face-to-face consultation). The caller descriptives were age (0–5, 6–18, 19–65, > 65 years), RFE (somatic illness, somatic injury, psychiatric illness, other), DOW (scale 1 to 5), profession of call-handler (nurse, physician, locum physician), day (weekday, weekend), and time of call (day = 08–16, evening = 16–24, night = 00–08). The triage response category “other” was excluded in statistical analyses due to small numbers (Intervention group: *n* = 392; control group: *n* = 379).

Descriptive analysis was performed using frequency distributions (numbers and percentages), median and interquartile range (IQR). The effect of DOW on triage response was analysed comparing intervention group and control group using a Chi-square test and a calculated odds ratio (OR). Results were reported as odds ratio’s (OR) with 95% confidence intervals (95%CI) and significance tests (*p* < .05). The modifying effect of age was assessed by stratified analyses for children aged 0–5 years and adults aged 65+ years (not presented).

Non-response bias was investigated comparing all calls (including the study population) during the data collection period with the study population concerning gender, age, RFE, and triage response. Data were analysed using SAS enterprise 7.12.

## Results

### Population

**Fig. 1:**
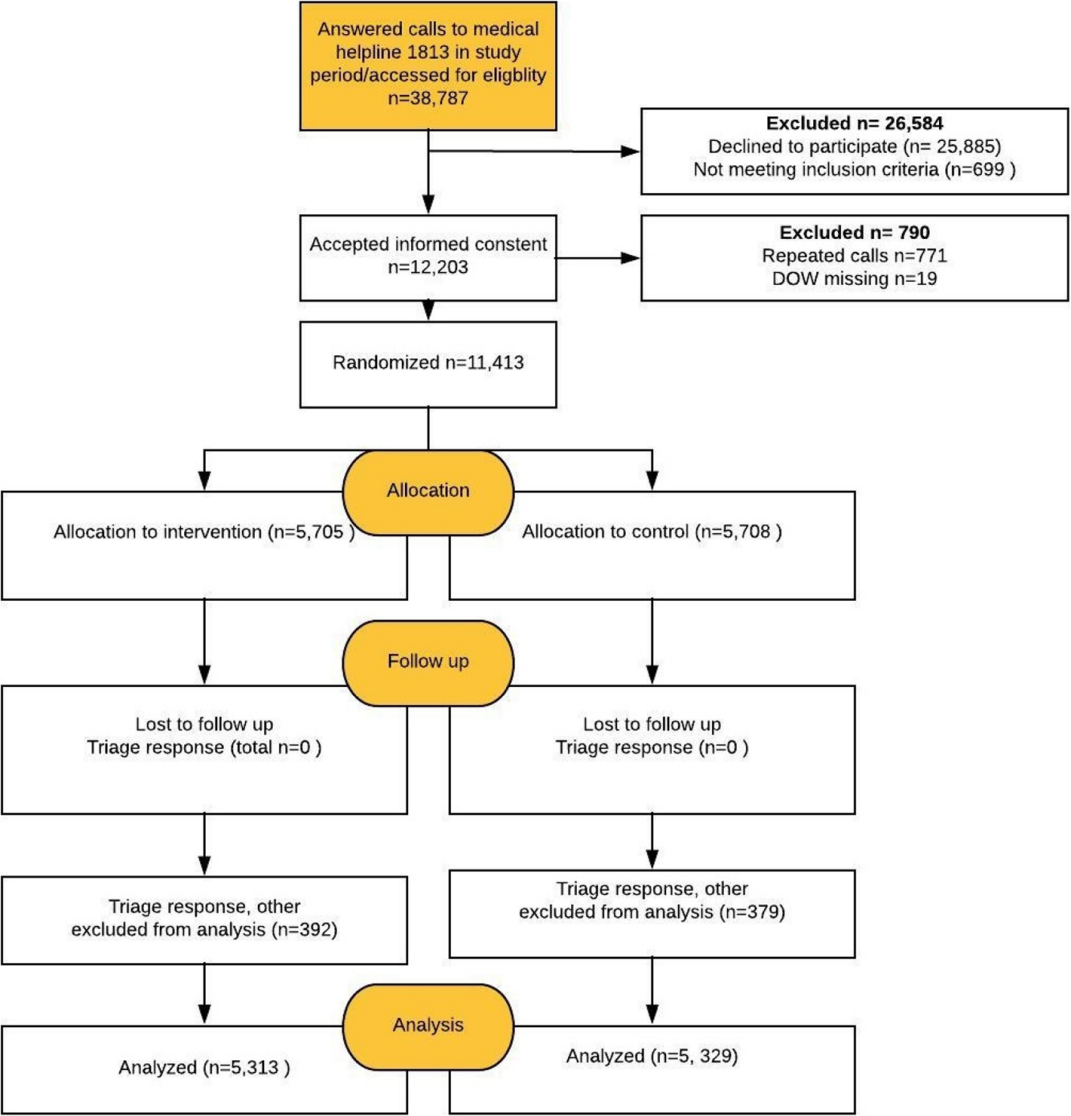
Flowchart of participants. Triage response “other” includes among others other guidance, answer on blood test, case summary after home visit

A total of 38,787 calls were received at MH1813 during the data collection period, of which 11,413 (33%) were randomized to either intervention group (*n* = 5705) or control group (*n* = 5708) after agreeing to participate (Fig. 1). The intervention group and control group were similar for caller, call and call-handler characteristics (Table 1). The study population had a mean age of 30.0 years (IQR = 6 to 49), 54.1% was female and DOW was normally distributed (DOW 1 = 9.5%, DOW 2 = 21.2%, DOW 3 = 36.0%, DOW 4 = 20.1%, DOW 5 = 13.3%). Telephone consultations were received by 43.8% of callers and face-to-face consultations by 49.5%. The distribution of RFE was: somatic illness 54.0%, somatic injury 18.1, and 0.9% psychiatric illness, while 24.0% were missing RFE. Most calls were made on weekdays (60.7%) and in the evening (53.8%), and calls were mostly handled by nurses (78.1%). The non-response analysis showed no difference between *all* calls during the data collection period and the study population for age, gender, RFE, and triage response (Additional file 4).

**Table 1:** Baseline demographic data for participating calls and call-handlers

	Intervention group *N* = 5705 N (%)	Control group *N* = 5708 N (%)
Gender, female	3077 (53.9%)	3101 (54.3%)
Age in years		
0–5	1326 (23.2%)	1292 (22.7%)
6–18	964(16.9%)	992 (17.4%)
19–65	2677 (46.9%)	2688 (47.1%)
66+	738 (12.9%)	732 (12.8%)
DOW		
1, minimally worried	521 (9.1%)	559 (9.8%)
2, a little worried	1210 (21.2%)	1207 (21.1%)
3, somewhat worried	2092 (36.7%)	2016 (35.3%)
4, very worried	1118 (19.6%)	1177 (20.6%)
5, extremely worried	764 (13.4%)	749 (13.1%)
Reason for encounter		
Somatic illness	3046 (53.4%)	3112 (54.5%)
Somatic injury	1044 (18.3%)	1021 (17.9%)
Psychiatric illness	24 (0.4%)	28 (0.5%)
Other^a^	223 (3.9%)	224 (3.9%)
Missing	1368 (24.0%)	1323 (23.2%)
Day		
Weekday	3463 (60.7%)	3468 (60.8%)
Weekend	2242 (39.3%)	2240 (39.2%)
Time of the day		
Day (8–16)	1889 (33.1%)	1920 (33.6%)
Evening (16–24)	3076 (53.9%)	3063 (53.7%)
Night (00–8)	740 (13.0%)	725 (12.7%)
Call-handler		
Nurse	4444 (77.9%)	4467 (78.3%)
Physician	1244 (21.8%)	1222 (21.4%)
Other (i.e. locum physician)	17 (0.3%)	19 (0%.3)

^a^Reason for calling “other” includes among others unintentional calls, calls from other regions, calls regarding transportation

### Triage response and DOW

The distribution of triage response was similar for the two groups: most callers received a face-to-face consultation (intervention: 53.6%, control: 52.5%), whereas 46.4% of the intervention group and 47.5% of the control group were triaged to telephone consultation (Table 2). The OR for getting a face-to-face consultation was 1.05 (95% CI: 0.98 to 1.14, *p* = 0.17). The same results were observed among young and older age groups (results not shown), the detailed triage response and at any time of day.

**Table 2:** Odds ratio for face-to-face consultation

Triage response^a^	Intervention group *N* = 5313 N (%)	Control group *N* = 5329 N (%)	OR (95% CI)
Telephone consultation	2463 (46.4)	2530 (47.5)	OR 1.05 (0.97–1.13)
Face-to-face consultation	2850 (53.6)	2799 (52.5)	

^a^The category of “other” is not included in the analysis (n = 392 intervention group vs. *n* = 379 control group)

### Process evaluation

The majority of the individuals staffing the call-handler stations during process evaluation were aware of the intervention and the placement of DOW on their screens (awareness 82–100%). Thirteen semi-structured interviews were conducted. The participating call-handlers mentioned that the placement of DOW was not sufficiently close to the main patient data (e.g. personal identification number, address) and they often missed the information. Some of the barriers voiced by the call-handlers were that the callers’ DOW could not be trusted, that the call-handlers intuitively recognized the callers’ DOW and that the provision of DOW was too big a responsibility to place on the caller. Moreover, many of the interviewed call-handlers believed that their intuition was as good as the scale to determine caller’s DOW, and therefore they did not consider DOW a useful parameter (Table 3).

**Table 3:** The thematic content of the process evaluation

Quotes from the study	Sub-themes	Themes
*I did not pay attention to the displayed DOW*	Structural problem in intervention	Intervention not received
*Another system was launched at the same time*		
*It’s a forest of information on the screens*	Information overload	
*I feel that I might annoy the caller asking about their DOW*	Barriers towards DOW	Intervention not delivered
*I think we place too much responsibility on the caller with the DOW scale*		
*By addressing something (DOW) we might leave out other questions*		
*By using the DOW, we omit our professional approach*		
*We already do this with our intuition*	DOW is intuitively recognized	
*We feel with our ears already*		
*I feel the worry in the intonation, speed of communication and choice of words*		
*I’m pretty new in the game and it helps me make the clinical decision*	DOW is useful in clinical decision-making	Usefulness of the scale
*The DOW makes an off set for the pedagogic part of the triage process*		
*It can support my telephone consultation but just that*	DOW is useful	
*It can be used as a safety net and can be used for obtaining more information*		

## Discussion

### Summary

We hypothesized that call-handlers’ awareness of the caller’s DOW would lead to less calls triaged to face-to-face consultation. However, we did not find a difference in triage response between intervention and control group. The process evaluation revealed that call-handlers were aware of the ongoing study and the location of the DOW, but that the placement of DOW on the screens was sub-optimal and sparsely used. Moreover, call-handlers voiced a concern that direct patient involvement in the telephone triage process could potentially place too much responsibility on the patient and that it would compromise the professional intuition.

### Strengths and limitations

The strength of the study is the design with random allocation of the intervention and complete follow-up of outcome measure. The setup of the data collection needed minimal effort from the intervention group and gave minimal recall bias concerning DOW. Even though a structural problem meant that DOW was not as easily visible on the call-handlers’ central screens as planned, which suggested that the intervention was compromised by changes in the computer display, the qualitative process evaluation revealed other barriers for incorporating DOW in the clinical decision process. These barriers most likely biased the results in the direction of no effect. The qualitative process evaluation was based on convenience sampling and another sampling strategy of participants might have changed the results. Moreover, only 33% of the callers participated in the study, which could result in selection bias. We might find more face-to-face responses within the non-respondent group, as they may feel not to have time due to a higher urgency and perhaps a higher DOW. This might imply that our findings cannot be generalized to the entire OOH patient population. However, our comparison of all callers with the study population did not detect differences on key characteristics including triage response.

### Comparison with existing literature

Our findings indicate that awareness of DOW does not have an impact on triage decision-making. Another explanation for our findings could be that the intervention was weak, as the process evaluation showed that the awareness and especially the use of DOW was limited. Although the staff was well informed of the study taking place, they did not pay attention to the displayed DOW. This could be ascribed to the placement of DOW on the computer screens and the relatively low likelihood of getting a caller from the intervention group (callers’ participation rate 33% and 1:1 allocation). Thus, call-handlers could not get a routine in using the DOW. Moreover, call-handlers expressed barriers towards incorporating DOW in their clinical decision-making process. The results might therefore be suffering from a type III error of an intervention not adequately implemented [25]. The main barriers voiced by the call-handlers were that the callers’ DOW could not be trusted, that the call-handlers intuitively recognized the callers’ DOW, and that it was too big a responsibility to place on the caller. These barriers towards patient participation are well known in healthcare [3, 26–28]. The issue of judging the callers credibility has been described before and might further serve as a barrier towards patient centred care in telephone triage [26, 29, 30].

#### Implications for research and practice

One other study on DOW in the MH1813 has found an association between DOW and triage response, [16] but this was a small study (*n* = 180). In a secondary analysis of DOW and illness perception, Thilsted et al. identified associations between the common sense model [31] and DOW [17]. Generally, a certain degree of over-triage is widely accepted in OOH telephone triage and, consequently, a large proportion of patients seen in face-to-face consultation are not considered medically relevant [31]. By integrating DOW in telephone consultation this proportion might be decreased since the question of DOW has been seen to open up for information sharing [16]. Moreover, as proposed by Kaminsky et al. another goal of telephone triage is prevention and education of the caller [32], which could be targeted by asking the question of DOW, because the response and advice could be directed to the callers’ concern. However, this will have to be investigated further.

## Conclusion

In this study, the awareness of callers’ DOW did not influence the calls-handlers’ triage response in a medical helpline. This finding might reflect a non-existing association or a weak implementation strategy resulting in insufficient use of the DOW. The process evaluation revealed several barriers towards incorporating the callers DOW in the clinical decision-making process. A repeated RCT with an improved design is needed to determine the effect of DOW on triage response. The literature supports a patient centred approach and the appliance of decision-tools in triage, and the use of DOW might help facilitate this.

## Additional files

Additional file 1: The flow of the electronic inclusion of participants. The call-handlers work-place consists of a table with a computer and four screens. The typical set-up of the screens from left to right is: 1) the desktop applications and telephone information on calls in queue, waiting time, and staff at work, access to websites on pharmacology treatment guidelines etc. 2-3) Two central screens, one displaying the patients’ basic information (e.g. address, personal identification number and dates of previous contacts to the medical helpline) and one having a template for current complaints and medical history. 4) the call-handler status (ready, working, recess) and the call-handler’s average talk-time and number of calls handled. (DOCX 196 kb)

Additional file 2: Call-handlers working station and screens. (DOCX 195 kb)

Additional file 3: Interview guide. (DOCX 16 kb)

Additional file 4: Descriptive of the full population with a valid personal identification number in the data collection period compared to the population. ^1^Triage response “other” includes among others other guidance, answer on blood test, case summary after home visit, request for prescription (DOCX 16 kb)
