# Supplementary figures and images for: Impact of caller’s degree-of-worry on triage response in out-of-hours telephone consultations: a randomized controlled trial
Source: Scand J Trauma Resusc Emerg Med. 2019 Apr 11;27:44. doi: 10.1186/s13049-019-0618-2 (PMC6458647; doi:10.1186/s13049-019-0618-2)

**Additional file 1.** The flow of the electronic inclusion of participants


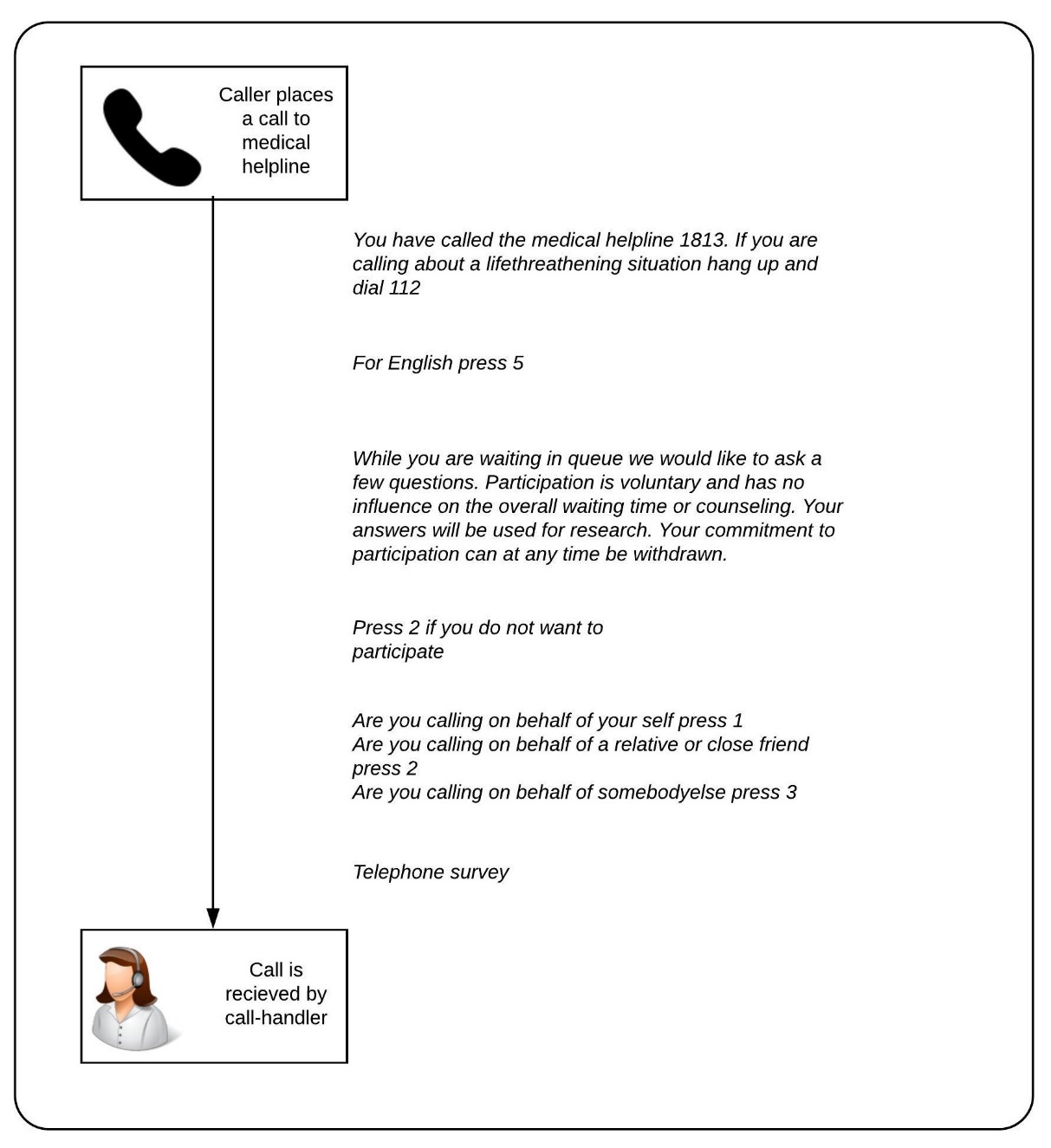

Supplement: Supplementary file 1 — The flow of the electronic inclusion of participants. The call-handlers work-place consists of a table with a computer and four screens. The typical set-up of the screens from left to right is: 1) the desktop applications and telephone information on calls in queue, waiting time, and staff at work, access to websites on pharmacology treatment guidelines etc. 2-3) Two central screens, one displaying the patients’ basic information (e.g. address, personal identification number and dates of previous contacts to the medical helpline) and one having a template for current complaints and medical history. 4) the call-handler status (ready, working, recess) and the call-handler’s average talk-time and number of calls handled. (DOCX 196 kb) [file 13049_2019_618_MOESM1_ESM.docx]
