# Supplementary material for: Impact of caller’s degree-of-worry on triage response in out-of-hours telephone consultations: a randomized controlled trial
Source: Scand J Trauma Resusc Emerg Med. 2019 Apr 11;27:44. doi: 10.1186/s13049-019-0618-2 (PMC6458647; doi:10.1186/s13049-019-0618-2)
Supplement: Supplementary file 3 — Interview guide. (DOCX 16 kb) [file 13049_2019_618_MOESM3_ESM.docx]

**Additional file 3.** Interview guide

- Do you always pay attention to the DOW when you take a call?
- What did you think/do when you saw that a caller had a DOW of 4-5?
- If you think back at a caller with a high DOW (4-5), can you then give me an example of this conversation and what you did?
- Did the DOW change your conversation with the caller? Explain.
- Has DOW changed your triage response to the individual caller? Explain.
- Does DOW mean anything to your work here? Why, why not?
- Is the usage of DOW something you have discussed with your colleges?
- Could you imagine yourself asking callers about their DOW?
- Do you think there would be any barriers about asking callers about their DOW?
