# Supplementary material for: Impact of caller’s degree-of-worry on triage response in out-of-hours telephone consultations: a randomized controlled trial
Source: Scand J Trauma Resusc Emerg Med. 2019 Apr 11;27:44. doi: 10.1186/s13049-019-0618-2 (PMC6458647; doi:10.1186/s13049-019-0618-2)
Supplement: Supplementary file 4 — Descriptive of the full population with a valid personal identification number in the data collection period compared to the population. 1Triage response “other” includes among others other guidance, answer on blood test, case summary after home visit, request for prescription (DOCX 16 kb) [file 13049_2019_618_MOESM4_ESM.docx]

**Additional file 4.** Descriptive of the full population with a valid personal identification number in the data collection period compared to the population

| **Callers** | **All calls**  N=38,787 (100%)  n (%) | **Study population**  N=11,413 (29.4%)  n (%) |
| --- | --- | --- |
| **Gender**  Female  Male | 21,248 (54.8)  17,538 (45.2) | 6,178 (54.1)  5,235 (45.9) |
| **Age in years**  0-5  6-18  19-65  66+ | 8,994 (23.2)  6236 (16.7)  19,270 (49.7)  4287 (11.1) | 2,622 (23.0)  1,956 (17.1)  5,365 (47.0)  1,470 (12.9) |
| **DOW**  1, minimally worried  2, a little worried  3, somewhat worried  4, very worried  5, extremely worried | N/A | 1,080 (9.5)  2,417 (21.2)  4,108 (36.0)  2,295 (20.1)  1,513 (13.3) |
| **Self-rated health**  1, very good  2, good  3, fair  4, bad  5, very bad  Missing | N/A | 2132 (18.9)  2766 (24.5)  2479 (21.9)  2230 (19.7)  1698 (15.0)  108 |
| **Reason for encounter**  Somatic illness  Somatic injury  Psychiatric illness  Other  Not registered | 20,726 (53.4)  6,554 (16.9)  217 (0.6)  2,017 (5.2)  9,273 (23.9) | 6,158 (54.0)  2,065 (18.1)  52 (0.5)  447 (3.9)  2,691 (23.6) |
| **Triage response**^1^  Telephone consultation  Face-to-face consultation  Other^1^ | 16,980 (43.8)  18,781 (48.4)  3,026 (7.8) | 4,993 (43.8)  5,649 (49.5)  771 (6.8) |
| *^1^ Triage response “other” includes among others other guidance, answer on blood test, case summary after home visit, request for prescription* | | |
